# Interaction between interleukin-10 (IL-10) polymorphisms and dietary fibre in relation to risk of colorectal cancer in a Danish case-cohort study

**DOI:** 10.1186/1471-2407-12-183

**Published:** 2012-05-17

**Authors:** Vibeke Andersen, Rikke Egeberg, Anne Tjønneland, Ulla Vogel

**Affiliations:** 1 Medical Department, SHS Aabenraa, DK-6200, Aabenraa, Denmark; 2 Institute of Regional Health Service Research, University of Southern Denmark, 5230, Odense, Denmark; 3 Medical Department, Viborg Regional Hospital, DK-8800, Viborg, Denmark; 4 Danish Cancer Society Research Center, DK-2100, Copenhagen, Denmark; 5 National Research Centre for the Working Environment, DK-2100, Copenhagen, Denmark; 6 National Food Institute, Technical University of Denmark, DK-2860, Soborg, Denmark; 7 Medical Department, Sygehus Sønderjylland Åbenrå, Egelund 10, DK-6200, Åbenrå, Denmark

**Keywords:** Gene-environment interaction, Dietary fibre, Fibers, Inflammation, Red and processed meat, Cereals, Fish, Carcinogenesis, Cohort, Prospective study, Population-based, Epidemiology, Smoking, NSAID

## Abstract

**Background:**

More than 50% of the colorectal cancer (CRC) etiology has been attributed to diet. Established or suspected dietary factors modifying risk of CRC are red meat, cereals, fish, and fibre. Diet and lifestyle may be linked to cancer through inflammation. Interleukin-10 (IL-10) is an anti-inflammatory cytokine. We wanted to test if dietary factors and *IL10* polymorphisms interact in relation to colorectal carcinogenesis.

**Methods:**

The functional *IL10* polymorphism C-592A (rs1800872) and the marker rs3024505 were assessed in relation to diet and lifestyle in a nested case-cohort study of 378 CRC cases and 775 randomly selected participants from a prospective study of 57,053 persons. Genotyping data on the *IL10* polymorphism C-592A, smoking and non-steroidal anti-inflammatory drugs (NSAID) was retrieved from Vogel et al. (Mutat Res, 2007; 624:88). Incidence rate ratios (IRR) and 95% Confidence Interval (95% CI) were calculated.

**Results:**

No associations were found between the *IL10* rs3024505 polymorphism and risk of CRC. There was interaction between rs3024505 and dietary fibre (P-value for interaction = 0.01). *IL10* rs3024505 homozygous wildtype carriers were at 27% reduced risk of CRC per 10 g fibre per day (95% CI: 0.60-0.88) whereas variant carriers had no risk reduction by fibre intake. Also, interaction between *IL10* C-592A and intake of fibre was found (P-value for interaction = 0.02). Among those eating <17.0 grams of fibre per day, carriers of an C-592A variant allele had a statistically significantly higher risk of colorectal cancer compared to homozygous wildtypes. No significant differences in colorectal cancer risk were observed between the reference group (CC and <17.0 g/day) and carriers of one C-592A variant allele eating 17.0 or more grams of dietary fibre per day. This suggests that the increased risk due to carrying the variant allele can be overcome by higher fibre intake. No interactions between *IL10* polymorphisms and dietary meat, cereal, or fish intake, or between *IL10* rs3024505 and smoking or NSAID use were found.

**Conclusions:**

In this northern Caucasian cohort we found interaction between *IL10* and dietary fibre in CRC carcinogenesis. High intake of fibre seems to protect against CRC among individuals with *IL10* related genetic susceptibility to CRC. This finding should be evaluated in other prospective and population-based cohorts with different ethnic groups.

## Background

Colorectal cancer (CRC) constitutes the second most common cancer in the Western World [1] and the burden of the disease is expected to increase dramatically due to demographic trends and implementation of westernized lifestyle in developing countries [2]. More than 50% of the CRC etiology has been attributed to diet and lifestyle [1,3] and, therefore, may be potentially avoidable by modification of these factors [4].

A modified inflammatory response may be an intermediate between diet and lifestyle on one hand and CRC on the other. Suspected dietary risk factors for CRC are red meat, dietary fibre, cereals and fish [4,5]. In a recent review, both intake of red meat and processed meat was linked to higher risk of CRC (summary effect estimate of 1.28 (95% CI 1.18-1.39) per 120 g red meat/day and 1.21 (95% CI: 1.04-1.42) per 50 g processed meat per day) [6]. Meat represents sources of pro-inflammatory n-6 poly-unsaturated fatty acids (PUFA), heterocyclic amines (HCA) and polycyclic aromatic hydrocarbons (PAH) formed during cooking at high temperature, haem iron and N-nitroso compounds [6], which may potentiate intestinal inflammation [7]. Dietary fibre were found to protect against CRC (adjusted relative risk 0.75 (95% CI: 0.59-0.95) for the highest versus lowest quintile of intake) [5]. Fibre are converted by colonic bacteria to short-chain fatty acids including butyrate which has been found to have anti-inflammatory properties [8]. Also cereal fibre has been shown to protect against colon cancer [9]. High fish intake has been found to protect against CRC [10,11] and fish is a source of anti-inflammatory n-3 PUFA [12]. Smoking has been reported to confer risk of CRC [13,14] and the mechanism may include induction of inflammation [15]. Long term use of non-steroidal anti-inflammatory drugs (NSAID) has been found to confer protection against CRC presumably by lowering an inflammatory response in colon [16,17].

Long term chronic inflammation is a well-established risk factor for CRC. Interleukin 10 (IL-10) [18,19] is an anti-inflammatory cytokine and crucial for maintaining intestinal homeostasis [20]. Animal studies suggest that insufficient IL-10 function promotes intestinal inflammation and CRC [21,22]. The biological mechanisms of action of IL-10 in the intestinal epithelium may involve blocking of the endoplasmic reticulum (ER) stress response (via inhibition of the ER activating transcription factor (ATF)-6 to the GRP78 promoter) [23] and, furthermore, inhibition of nuclear factor-κB (NFκB) activation (via stabilisation of inhibitory-κB (IκB)) [24]. Diet and lifestyle may directly or indirectly affect IL-10 function. For example, in animal models, mucosal IL-10 production has been found to be stimulated by intestinal microbes [25,26] which, on the other hand, have been found to be affected by diet and NSAID use [27,28]. Also, studies on isolated leukocytes showed that fatty acids stimulate the production of IL-10 [29].

Investigating functional genetic variants in carefully selected candidate genes in combination with diet and life-style factors in well-characterized prospectively collected study populations has previously been successfully applied for the study of underlying carcinogenic mechanisms of food items [30-32]. The functional *IL10* promoter polymorphisms G-1082A, C-819 T, and C-592A are in tight linkage disequilibrium. The haplotype encompassing all three variant polymorphisms is associated with low IL-10 protein production in lymphocytes *in vitro*[33] and *in vivo*[34,35] probably because the A allele of the *IL10* promoter polymorphism C-592A leads to the formation of a binding site for the ETS family of transcription factors [36]. An association between the low activity-associated *IL10* C-592A and *IL10* G-1082A variant genotypes and high risk of CRC has been found in some case–control studies [37-39] but not all [40]. Another polymorphism, the marker rs3024505 immediately downstream of the *IL10* gene, has not been investigated in relation to CRC but has been associated with risk of inflammatory bowel disease [41,42].

Hence, in search for a biological understanding, we hypothesized that dietary components and lifestyle may modify CRC risk through modifying the inflammatory response in colon by modifying IL-10 expression. An interaction between functional polymorphisms in *IL10* and diet would indicate that IL-10 and hence the inflammatory response is implicated in the pathway leading from the dietary component to CRC. We therefore investigated the interaction between the functional polymorphism *IL10* C-592A and the marker rs3024505 and diet, and between rs3024505 and smoking and NSAID use in relation to development of CRC in a case-cohort study of 378 CRC cases and 775 randomly selected participants from the prospective population-based Danish Diet, Cancer and Health study.

## Methods

### Studied subjects

Diet, Cancer and Health is an ongoing Danish cohort study designed to investigate the relation between diet, lifestyle and cancer risk [43]. The cohort consists of 57,053 persons, recruited between December 1993 and May 1997. All the subjects were born in Denmark, and the individuals were 50 to 64 years of age and had no previous cancers at study entry. Blood samples and questionnaire data on diet and lifestyle were collected on study entry.

### Follow-up and endpoints

Follow-up was based on population-based cancer registries. Between 1994 and 2003, 405 CRC cases (184 women and 221 men) were diagnosed. A subcohort of 810 persons (368 women and 442 men) were randomly selected within the cohort [38,43]. Blood samples were available for 397 cases and 800 subcohort members. All information on genotypes and diet and lifestyle factors was available for 378 cases and 775 subcohort members (44 with missing genotype data and 18 with missing diet and/or lifestyle data were excluded).

### Dietary and lifestyle questionnaire

Information on diet, lifestyle, weight, height, medical treatment, environmental exposures, and other socio-economic factors were collected at enrolment.

In the food-frequency questionnaire, diet consumption was assessed in 12 categories of predefined responses, ranking from’never’ to’eight times or more per day’. The daily intake was then calculated by using FoodCalc [44]; this program uses population specific standardized recipes and portion sizes. Intake of red meat in grams per day was calculated by adding up intake of beef, veal, pork, lamb and offal. Intake of processed meat in grams per day was calculated by adding up intake of processed red meat, including bacon, smoked ham, salami, frankfurter, Cumberland sausage, cold cuts and liver pâté.

Dietary fibre intake were based on country-specific food composition tables, which were reviewed to ensure comparability to the association of official analytical chemists (AOAC) fibre definition, which includes lignin and resistant starch [45]. Fibre intake is calculated by multiplying the frequency of consumption of relevant foods by their fibre content as determined from national databases of food content [46].

Contributing food items to the food group ‘cereals’ included wholegrain foods (wholegrain bread, rye bread, wholegrain flour, oatmeal, corncobs, müsli, and crispbread) and refined grain foods (white wheat bread, wheat flour, rice flour, potato flour, corn flour/starch, pasta, wheat) and was measured in grams per day [47]*.*

Intake of ‘fish’ in grams per day was calculated by adding up intake of fresh and processed fish.

The questionnaire was tested in a pilot study preceding the Diet, Cancer and Health study. Pearson correlation coefficients (adjusted for total energy intake) illustrating the comparison of nutrient scores estimated from the food-frequency questionnaire and from weighed diet records were 0.39 and 0.53 for dietary fibre and 0.37 and 0.14 for meat for men and women, respectively [48,49].

Smoking status was classified as never, past or current.

The lifestyle questionnaire included this question regarding use of NSAID: “Have you taken more than one pain relieving pill per month during the last year?” If the answer was yes, the participant was asked to record how frequently they took each of the following medications: “Aspirin”, “Paracetamol”, “Ibuprofen”, or “Other pain relievers”. The latter category included NSAID preparations other than aspirin and ibuprofen. Based on all records, we classified study subjects according to use of “any NSAID” (≥ 2 pills per month during one year) at baseline.

### Genotyping

Buffy coat preparations were stored at minus 150^0^ C until use. DNA was extracted as described [50]. Genotyping was performed in 5 ul reactions containing 2.5 ul mastermix (Applied Biosystems), ca 20 ng DNA and 1 mM primers and 100 nM probes or 1 x predeveloped assay. *IL10* rs3024505 were determined using the pre-developed allelic discrimination assay C_15869717_10 (Applied Biosystems). C-592A (rs1800872) was determined and reported previously [38]. Primers were: Forward: 5′-GGTAAAGGAGCCTGGAACACATC-3′, Reverse: 5′-CCAAGCAGCCCTTCCATTT-3′ and probes were: A-allele: 5’- VIC-ACCCCGCCTGTACTGTAGGAAGC-TAMRA-3′ and C-allele: FAM-ACCCCGCCTGTCCTGTAGGAAGC-TAMRA-3′ [51].

Laboratory staff was blinded to case/control status during analysis. Known genotype controls were included in each run. To confirm reproducibility, genotyping was repeated for 10% of the samples yielding 100% identity.

### Statistical analysis

Hardy-Weinberg equilibrium was determined manually by the χ^2^−test.

The analyses were performed according to the principles for the analysis of case-cohort studies [52]. The analyses were performed unweighted. Age was used as the time scale in the Cox regression model. Tests and confidence intervals were based on Wald’s test using the robust estimate of the variance-covariance matrix for the regression parameters in the Cox regression model [53].

All models were adjusted for baseline values of suspected risk factors for colorectal cancer such as body mass index (BMI) (kg/m^2^, continuous), NSAID (yes/no), use of hormone replacement therapy (HRT) (never/past/current, among women), smoking status (never/past/current), intake of dietary fibre (g/day, continuous), and red meat and processed meat (g/day, continuous).

The likelihood ratio test was used for interaction analyses between the two *IL10* polymorphisms and intake of meat, dietary fibre, cereals, fish, and between rs3024505 and smoking status and NSAID use. In interaction analyses between *IL10* polymorphisms and dietary fibre intake entered as a categorical variable, tertile cutpoints were based on the distribution among cases. Trend test were calculated using the Wald test.

All analyses were performed using SAS version 9.1 (SAS Institute Inc., Cary, NC).

A p < 0.05 was considered to be significant.

### Ethics

All participants gave verbal and written informed consent. The study was approved by the regional Ethics Committees on Human Studies (Jr.nr. (KF)11-037/01 and jr.nr. (KF)01-045/93), and the Danish Data Protection Agency.

## Results

Characteristics of the study population and risk factors for CRC are shown in Table 1. There were significant differences in baseline characteristics between the cases and subcohort members for age and intake of fibre (Table 1). The genotype distribution of the polymorphisms in the subcohort did not deviate from Hardy-Weinberg equilibrium (results not shown). The variant allele frequency for *IL10* rs3024505 and C-592A were 0.16 and 0.22, respectively, in the subcohort.

**Table 1:** Baseline characteristics of study population selected for the diet, cancer and health cohort

	**Cases**		**Subcohort**		**P-value**
	**No. (%)**	**Medians (5-95%)**	**No. (%)**	**Medians (5-95%)**	
Total	378 (100)		775 (100)		
Sex					
Men	211 (33)		422 (67)		
Women	167 (32)		353 (68)		
Age at inclusion (*years*)		58 (51–64)		56 (50–64)	<0.0001
BMI (*kg/m*^*2*^)		26 (21–34)		26 (20–33)	0.06
Food intake (*g/day*)					
Alcohol^1^		15 (1–68)		13 (1–63)	0.05
Dietary fibre		20 (10–31)		21 (11–35)	0.001
Red and processed meat		111 (50–240)		109 (40–239)	0.73
Smoking status					0.47
Never	118 (31)		261 (34)		
Past	115 (30)		245 (32)		
Current	145 (38)		269 (35)		
NSAID use					0.53
No	255 (67)		537 (69)		
Yes	123 (33)		238 (31)		
HRT use among					0.71
women	97 (26)		187 (24)		
Never	26 (7)		61 (8)		
Past	44 (12)		105 (14)		
Current					

^1^ Among current drinkers.

### Associations between polymorphisms and CRC

There was no association between *IL10* rs3024505 genotypes and CRC risk (Table 2). C-592A was genotyped and the association to risk of CRC was reported previously [38]. Since we observed no gene-dose effects, variant genotypes were combined in the interaction analysis to maximize the statistical power. Haplotype distribution among the controls showed no linkage between the two polymorphisms and therefore haplotype analysis was not performed.

**Table 2:** **Incidence rate ratios and 95% confidence intervals for the studied *IL10 *** **gene polymorphisms in the diet, cancer and health study**

	**N**_**Case**_	**N**_**Subcohort**_	**Crude IRR (95% CI)**^**a**^	**Adjusted IRR (95% CI)**^**b**^	**P-value**^**c**^
**rs3024505**					
CC	268	553	1.00	1.00	-
CT	99	202	0.99 (0.79-1.25)	1.01 (0.80-1.28)	0.91
TT	11	20	1.15 (0.63-2.10)	1.11 (0.61-2.03)	0.73
CT and TT	110	222	1.01 (0.81-1.26)	1.02 (0.82-1.28)	0.85
**C-592A** (rs1800872)^**d**^					
CC	238	470	1.00	1.00	-
AC	116	261	0.91 (0.73-1.14)	0.90 (0.72-1.12)	0.36
AA	24	44	1.11 (0.74-1.67)	1.10 (0.73-1.65)	0.65
AC and AA	140	305	0.94 (0.77-1.16)	0.93 (0.76-1.14)	0.50

^a^ Crude analyses.

^b^ Analyses adjusted for smoking status, alcohol, HRT status (women only), BMI, use of NSAID, and intake of red and processed meat and dietary fibre.

^c^ P-value for the adjusted estimates.

^d^Data for *IL10* rs1800872 were taken from (38).

### Gene-environmental analyses

There was interaction between rs3024505 and intake of dietary fibre (P-value for interaction = 0.01) (Table 3). *IL10* rs3024505 homozygous wildtype carriers were at 27% reduced risk of CRC per 10 g fibre per day (95% CI: 0.60-0.88) whereas variant carriers had no risk reduction by fibre intake. C-592A variant allele carriers were at reduced risk of CRC by fibre intake (IRR pr 10 g fibre/day: 0.71 (0.54-0.93)) whereas homozygous wildtype allele carriers were not (IRR pr 10 g/day: 0.90 (0.74-1.09)). There was however no statistically significant interaction between genotype and fibre intake (P-value for interaction = 0.15) (Table 3). No statistically significant interactions between *IL10* polymorphisms and dietary meat, cereals, or fish intake were found (Table 3).

**Table 3:** **Interaction between dietary factors and the studied *IL10 *** **polymorphisms in relation to colorectal cancer risk**

	**Crude IRR (95% CI)**^**a**^	**Adjusted IRR (95% CI)**^**b**^	**P**_**Interaction**_^**c**^
	*Intake of red and processed meat (per 25 g/day)*		
**rs3024505**			
CC	1.02 (0.96-1.07)	1.02 (0.96-1.08)	
CT and TT	1.02 (0.96-1.09)	1.03 (0.96-1.10)	0.78
**C-592A** (rs1800872)			
CC	1.01 (0.96-1.06)	1.02 (0.97-1.07)	
AC and AA	1.03 (0.96-1.11)	1.02 (0.95-1.11)	0.92
	*Intake of dietary fibre (per 10 g/day)*		
**rs3024505**			
CC	0.70 (0.59-0.84)	0.73 (0.60-0.88)	
CT and TT	1.10 (0.83-1.46)	1.12 (0.84-1.49)	0.01
**C-592A** (rs1800872)			
CC	0.88 (0.73-1.06)	0.90 (0.74-1.09)	
AC and AA	0.67 (0.51-0.88)	0.71 (0.54-0.93)	0.15
*Intake of cereals (per 50 g/day)*			
**rs3024505**			
CC	0.89 (0.82-0.97)	0.99 (0.88-1.12)	
CT and TT	1.03 (0.91-1.16)	1.13 (0.98-1.30)	0.09
**C-592A** (rs1800872)			
CC	0.95 (0.87-1.04)	1.05 (0.93-1.18)	
AC and AA	0.91 (0.81-1.02)	1.01 (0.88-1.16)	0.63
*Intake of fish (per 25 g/day)*			
**rs3024505**			
CC	0.88 (0.78-1.00)	0.89 (0.79-1.01)	
CT and TT	0.95 (0.76-1.20)	0.97 (0.77-1.24)	0.51
**C-592A** (rs1800872)			
CC	0.85 (0.74-0.97)	0.85 (0.74-0.98)	
AC and AA	1.01 (0.84-1.21)	1.03 (0.86-1.24)	0.09

^a^ Crude analysis.

^b^ Analysis adjusted for smoking status, alcohol, HRT status (women only), BMI, use of NSAID, and intake of red and processed meat and dietary fibre.

^c^ P-value for interaction between polymorphisms and dietary factors for the adjusted estimates.

Because of the interaction between rs3024505 and intake of dietary fibre, tertile analysis of fibre intake and genotype in relation to CRC was performed to obtain more information about the association of the studied polymorphisms in absence of fibre intake. Among those eating <17.0 grams of fibre per day, carriers of the C-592A variant allele had a statistically significantly increased risk of colorectal cancer compared to homozygous wildtype carriers. Interestingly, no significant differences in CRC risk were observed between the reference group (CC and <17.0 g/day) and carriers of the C-592A variant allele eating 17.0 or more grams of dietary fibre per day (Table 4). This suggests that the increase in risk associated with carrying the variant allele could be overcome by higher fibre intake.

**Table 4:** **Incidence rate ratio (IRR) for colorectal cancer for tertiles of intake of dietary fibre by *IL10 *** **polymorphisms among cases (N**_**c**_**) and subcohort (N**_**s**_**)**

	**Intake of dietary fibre**						**IRR (95% CI)**^**a**^			
	**1.tertile**		**2.tertile**		**3.tertile**		**Intake tertiles (g/day)**			
	**N**_**c**_	**N**_**s**_	**N**_**c**_	**N**_**s**_	**N**_**c**_	**N**_**s**_	**<17.0**	**17.0-22.7**	**>22.7**	**P**_**Int**_^**b**^
**rs3024505**										
CC	96	152	86	176	86	225	1.00	0.87 (0.65-1.16)	0.73 (0.55-0.98)	
CT and TT	28	56	39	82	43	84	0.83 (0.54-1.27)	0.83 (0.58-1.18)	0.96 (0.66-1.38)	0.16
**C-592A** (rs1800872)										
CC	69	137	81	148	88	185	1.00	1.17 (0.84-1.61)	1.08 (0.78-1.49)	
AC and AA	55	71	44	110	41	124	1.44 (1.02-2.05)	0.87 (0.59-1.27)	0.80 (0.54-1.19)	0.02

^a^ Analysis adjusted for smoking status, alcohol, HRT status (women only), BMI, use of NSAID, and intake of red and processed meat.

^b^ P-value for interaction between polymorphisms and dietary factors for the adjusted estimates.

When the two genotypes were combined, fibre intake was associated with the most protective effect among homozygous wildtype carriers of rs3024505 who were variant allele carriers of C-592A (IRR = 0.55; 95% CI: 0.35-0.86) (Table 5). The observed pattern suggested additive effects of the two polymorphisms and there was no statistically significant interaction (P-value for interaction = 0.19).

**Table 5:** Risk of CRC in relation to fibre intake subdivided by * IL10 * genotype combinations

	**Crude** ** IRR (95% CI)**^**a**^	**Adjusted** ** IRR (95% CI)**^**b**^	**P**_**Interaction**_^**c**^
	*Intake of dietary fibre (per 10 g/day)*		
**rs3024505/C-592A** (rs1800872)			
wt/wt	0.64 (0.43-0.93)	0.65 (0.44-0.96)	
wt/variant	0.53 (0.34-0.83)	0.55 (0.35-0.86)	
variant/variant	0.72 (0.36-1.44)	0.75 (0.38-1.47)	
variant/wt	1.18 (0.86-1.62)	1.19 (0.87-1.65)	0.19

^a^ Crude analysis.

^b^ Analysis adjusted for smoking status, alcohol, HRT status (women only), BMI, use of NSAID, and intake of red and processed meat and dietary fibre.

^c^ P-value for interaction between polymorphisms and dietary factors for the adjusted estimates.

No statistically significant interactions between *IL10* rs3024505 polymorphism and smoking (results not shown) or NSAID use (results not shown) was found.

## Discussion

In the present study, we found interaction between the *IL10* polymorphisms and intake of dietary fibre. High intake of fibre was associated with lowered risk of CRC among the *IL10* rs3024505 homozygous wildtype allele carriers, but not among variant allele carriers (P-value for interaction = 0.01). Furthermore, in tertile analyses, risk of CRC was reduced among C-592A variant allele carriers by high fibre intake compared to wildtype allele carriers who had no risk reduction by fibre intake (P-value for interaction = 0.02). No statistically interactions between *IL10* polymorphisms and dietary meat, cereals, or fish intake or between *IL10* rs3024505 polymorphism and smoking or NSAID use were found in relation to CRC.

The protective effect of dietary fibre on risk of CRC is well documented [5]. To our knowledge, this is the first study which suggests that the protective effect of dietary fibre may involve an interaction with anti-inflammatory regulation. Fibre are the indigestible portion of plant foods whereof the soluble fibre are fermented by bacteria in the digestive tract and the insoluble fibre have bulking action. Of particular interest, dietary intake of starch have been found to impact the composition of the gut microbiota [54]. Commensal bacteria such as *Clostridia*-related species and *Bacteroides fragilis* have been shown to affect host IL-10 responses and result in enhanced resistance to intestinal infection in mouse models [55-58]. Similarly, increased IL-10 level in blood was found after 4 weeks intake of dietary fibre in overweight humans [59]. Our results support the notion of biological interaction between fibre intake and host IL-10 synthesis. Furthermore, our results suggest this has beneficial effects in individuals carrying the C-592A variant allele and/or two alleles of rs3024505.

A case–control study of 495 gastric cancer (GC) cases and 495 controls found no interaction between smoking and *IL10* genotypes [60]. Both carriers of the C-592A homozygous wildtype genotype and carriers of the variant genotypes had high risk of GC among smokers (odds ratio (OR) 2.88 (95% CI: 1.67-4.95) and 2.06 (95% CI: 1.20-3.55), respectively) compared to the non-smoking reference group (p for interaction = 0.541) [60]. No other studies on *IL10* polymorphisms and dietary fibre, meat, cereals, or fish intake, smoking status or NSAID use in relation to CRC or any other cancer were found.

The present study found no association between *IL10* rs3024505 polymorphism *per se* and risk of CRC. This polymorphism is a marker polymorphism with no known function which has been associated with IBD but which has not been studied in relation to CRC [41,42]. Associations between the low activity-associated *IL10* C-592A and *IL10* -1082 (rs1800896) variant genotypes and high risk of CRC have been found by some case–control studies [37-39] but not all [40]. Thus, carriers of the C-592A homozygous variant genotype were at high risk of CRC compared to the homozygous wildtype carriers in the studies by Čačev et al. and Vogel et al. (odds ratio (OR) of 4.07 (95% CI: 1.28-12.96) and OR 1.25 (95% CI: 0.72-2.18), respectively) [37,38]. *IL10* has been studied in relation to other cancers, including gastrointestinal cancers [61-66], however, the results have been ambiguous. *IL10* has not been associated with CRC in GWAS [67,68]. Our study suggests that intake of fibers may play a role in individuals carrying the C-592A variant allele and in individuals carrying the rs3024505 homozygous wildtype genotype. In the combination analysis of the two polymorphisms, additive effects were found, suggesting that both polymorphisms contribute to the found effects. Our results suggest that *IL10* polymorphisms may be most important in populations with low fibre intake.

This study used a nested case-cohort design which is efficient and has the major advantage that data and samples were collected before diagnosis. A main strength of our study is a well-characterized study population. Information on diet and lifestyle factors was collected at enrolment for all participants which minimizes the risk of differential misclassification of cases and comparison group. However, data were only collected once, and may thus not be representative for the lifestyle during follow-up. This is, however, not expected to result in differential misclassification. Furthermore, information on food intake was based on a semi-quantitatively food frequency questionnaire [43,49], which was evaluated and found usable [48]. In addition, the results were adjusted for known confounding factors affecting the risk of CRC in this cohort including dietary factors, body mass index (BMI), alcohol, smoking status and NSAID use. Cases were generally older than the comparison group at inclusion into the Diet, Cancer and Health cohort. This is because the comparison group was chosen as a random sample of the study cohort, whereas cases were defined by their age at diagnosis. However, care was taken to ensure that all risk estimates were age adjusted and this means that we generally had longer follow up on the members of the comparison group than of the cases, because the CRC cancer cases generally belonged to the older age fraction of the recruited persons. Fibre intake was lower among the cases compared to controls. To prevent confounding, all risk estimates were adjusted for fibre intake. A main limitation of the study is the limited sample size.

A power calculation showed that we had 82% chance of detecting an OR of 1.5 [69]. Therefore, since no gene dose effects were observed, heterozygous and homozygous variant genotype carriers were combined for the analyses of interactions to obtain sufficient statistical power. Nevertheless, in the light of the obtained P-values and the number of statistical tests performed, we cannot exclude that our positive findings may be due to chance. Moreover, we cannot exclude that associations and/or interactions may not have been identified due to insufficient power.

The present result is in agreement with previous studies proving the concept that gene-environmental interactions may be utilized for identification of underlying biological pathways in carcinogenesis [31,32]. The mentioned studies suggested that the intestinal transporter ABCB1 and nuclear factor kappa B (NFκB) are involved in meat carcinogens.

## Conclusions

This study of a Northern Caucasian cohort suggests interaction between *IL10* and dietary fibre in CRC carcinogenesis. High intake of fibre seems to protect against CRC among individuals with *IL10* related genetic susceptibility to CRC. This finding should be evaluated in other prospective population-based cohorts where information on fibre intake is available.

## Abbreviations

BMI: Body mass index; CRC: Colorectal cancer; HRT: Hormone replacement therapy; NSAID: Non-steroidal anti-inflammatory drugs; IL-10: Interleukin 10; CI: Confidence interval; IRR: Incidence rate ratio.

## Competing interests

The authors declare that they have no competing interests.

## Authors' contributions

VA and UV designed the molecular genetic studies, UV determined the genotypes and VA drafted the manuscript. AT designed the cohort and RE performed the statistical analysis. VA and UV conceived of the study, its design and coordination. All authors helped to draft the manuscript, read and approved the final manuscript.

## Pre-publication history

The pre-publication history for this paper can be accessed here:

http://www.biomedcentral.com/1471-2407/12/183/prepub
